# Dairy consumption, bone turnover biomarkers, and osteo sono assessment index in Japanese adults: A cross-sectional analysis of data from the Iwaki Health Promotion Project

**DOI:** 10.1016/j.bonr.2024.101770

**Published:** 2024-04-29

**Authors:** Ayatake Nakano, Hiroshi M. Ueno, Daisuke Kawata, Yota Tatara, Yoshinori Tamada, Tatsuya Mikami, Koichi Murashita, Shigeyuki Nakaji, Ken Itoh

**Affiliations:** a Milk Science Research Institute, Megmilk Snow Brand Co., Ltd., Kawagoe, Japan; b Department of Precision Nutrition for Dairy Foods, Hirosaki University Graduate School of Medicine, Hirosaki, Japan; c Research Center for Health-Medical Data Science, Hirosaki University Graduate School of Medicine, Hirosaki, Japan; d Innovation Center for Health Promotion, Hirosaki University Graduate School of Medicine, Hirosaki, Japan; e Research Institute of Health Innovation, Hirosaki University, Hirosaki, Japan; f Center for Advanced Medical Research, Hirosaki University Graduate School of Medicine, Hirosaki, Japan

**Keywords:** Dairy, Bone turnover markers, Osteo sono assessment index, Japan, Cross-sectional study

## Abstract

**Purpose:**

Dairy foods are nutritional sources of calcium, phosphorus, protein, and other nutrients that improve bone health. However, the effects of dairy consumption on bone biomarkers in the Japanese population remain unclear. This study explored the association between dairy consumption and bone biomarkers in Japanese adults.

**Methods:**

This cross-sectional study was conducted as part of the Iwaki Health Promotion Project in Aomori, Japan. In total, 1063 adults were included in the analysis. Bone turnover marker levels were measured in local citizens during their annual medical checkups. The calcaneus osteo sono assessment index (OSI) was calculated using a quantitative ultrasound technique. The dietary intake of foods and nutrients was estimated using a food frequency questionnaire. Linear regression models were established using dairy consumption and bone biomarkers with adjustments. Statistic significance was considered by *P* < 0.05.

**Results:**

In multivariate models, the tartrate-resistant acid phosphatase 5b and parathyroid hormone concentrations were inversely associated with dietary dairy consumption after adjusting for age and sex. The undercarboxylated osteocalcin, a procollagen type I N-terminal peptide to bone alkaline phosphatase ratio, and OSI were the directly associated with dairy consumption in multivariate models with adjustment.

**Conclusions:**

Dairy consumption is partially associated with bone turnover biomarkers and OSI in adult Japanese participants. Habitual consumption of dairy foods may contribute to the nutritional supplementation for maintaining bone health, including turnover and structure.

**Clinical trial registry number and website where it was obtained:**

The Japanese Clinical Trials Registry (UMIN000040459), https://center6.umin.ac.jp/cgi-open-bin/ctr/ctr_view.cgi?recptno=R000046175.

## Highlights

• We explored the association between dairy consumption and bone biomarkers in Japan.
• 1063 adults (mean age, 54 years; 62 % female) were included in the analysis
• Dairy consumption was partially associated with bone turnover biomarkers and OSI.
• Habitual consumption of dairy foods may contribute to maintaining bone health.

## 1 Introduction

High consumption of dairy foods is associated with bone health and related health outcomes, such as hip fractures (Bian et al., 2018). Milk and dairy are balanced dietary sources of essential and nonessential nutrients. Essential nutrients from dairy foods include proteins, fats, carbohydrates, and micronutrients, such as calcium, potassium, and vitamin D (Haug et al., 2007; Rizzoli, 2022). Nonessential nutrients vary among dairy foods, such as probiotics, prebiotics, parabiotics, and postbiotics from fermented dairy products, bioactive proteins from fractionated milk protein, and bioactive peptides from enzymatic hydrolysates of milk protein (García-Burgos et al., 2020; Park and Nam, 2015). However, the detailed association is inconclusive.

The Japanese diet comprises a mixture of *washoku*, a traditional Japanese diet, and a Westernized diet (Shirota et al., 2022; Park et al., 2023). The traditional Japanese diet consists of rice, soybeans, seafood, and vegetables in different preparation methods, including fermentation. The modern Japanese diet is characterized by low consumption of milk and cheese and comparable consumption of yoghurt worldwide (Miller et al., 2022).

The importance remains unclear in the relationship between dairy consumption and biomarkers of bone health in community-dwelling adults in Japan. Bone turnover markers are evaluated to estimate fracture risk in individuals affected by bone loss and are classified as bone formation or resorption (Vasikaran et al., 2011; Kuo and Chen, 2017). Osteoporosis is characterized by low bone mass and deterioration of the bone microarchitecture in bone tissue, along with a consequent increase in bone fragility and susceptibility to fractures (Compston et al., 2019). Prevention of osteoporosis is emphasized in postmenopausal females because of the first established link between bone loss and decreased estrogen levels (Norman and Henry, 2015; Meng-Xia and Qi, 2015). Japan is one of the super-aged society and the high longevity in elderly females, and we hypothesized that even the low dairy consumption potentially influenced the bone biomarkers.

In this study, we aimed to determine the bone formation and resorption markers and the osoteo sono assessment index (OSI) profile in a community-based Japanese population including postmenopausal females and explore the associations between dairy consumption and these bone turnover markers. The associations were further examined in postmenopausal females and treatment status of osteoporosis.

## 2 Methods

### 2.1 Ethics approval

The study protocol was approved by the Internal Review Board of Hirosaki University (ref. #2014-377-1 and 2022-121) and registered in the Japanese Clinical Trials Registry (UMIN000040459). This study was conducted in accordance with the 1975 Declaration of Helsinki, revised in 1983. Written informed consent was obtained from all the participants at the approval of the original study protocol in the Iwaki Health Promotion Project (IHPP) 2015, followed by completing the opt-out procedure in March 2023.

### 2.2 Participants and samples

A cross-sectional study was conducted as part of the IHPP in Aomori Prefecture, Japan (Nakaji et al., 2021; Mikami et al., 2020). The IHPP was coordinated as a social innovation platform for health promotion. We recruited healthy adult residents of the Iwaki Region of Hirosaki City in northern mainland Japan during an annual medical checkup in June 2015. The medical checkup was conducted over 10 consecutive days, and the participants voluntarily participated after a public announcement. The inclusion criteria were healthy adults (age ≥ 20 years) willing to participate the medical checkup and provide written informed consent for the IHPP conducted in 2015 and subsequent opt-out procedure for this study. The exclusion criterion was missing data for each analysis's dependent and independent variables.

According to the study protocol's telephone and documented instructions, the participants underwent a series of medical checkups, including questionnaire completion, physical and written examinations, and blood sample assessment. Whole blood samples were obtained after overnight fasting and subjected to laboratory tests.

A total of 1113 participants were eligible from the source cohort, and 1063 participants (407 men and 656 females) were analyzed in this study after exclusion due to missing data on diet and bone biomarkers. In addition, 29 participants received treatment for osteoporosis. A flowchart of the disposition and grouping of the study participants is shown in Supplementary Fig. 1.

### 2.3 Bone turnover biomarkers

In the present study, we adopted six bone turnover markers and the calcaneus osteo sono assessment index (OSI) T-score and Z-score from the source cohort. The bone turnover markers were classified as either markers of bone formation, including total procollagen type 1 N-terminal propeptide (P1NP), bone-specific alkaline phosphatase (BAP), and intact PTH, or bone resorption markers, including N-terminal cross-linked telopeptide of type 1 collagen (NTx), tartrate-resistant acid phosphatase-5b (TRACP-5b), and undercarboxylated osteocalcin (ucOC). The serum total P1NP and BAP levels were determined using electrochemiluminescence immunoassay and chemiluminescent enzyme immunoassay (EIA), respectively. Serum levels of NTx and TRACP-5b were determined using EIA. All laboratory tests were performed in a commercial laboratory (LSI Medience, Tokyo, Japan). To evaluate bone metabolism, bone formation and resorption indices were calculated as the P1NP-to-BAP and NTx-to-TRACP-5b, respectively (Mikami et al., 2020).

### 2.4 Osteo sono assessment index

The OSI T-score was determined as described in our previous study with minor modifications (Mikami et al., 2020). Briefly, the OSI of the calcaneus was measured using quantitative ultrasound (QUS) with AOS-100NW (Hitachi, Ltd., Tokyo). The results were calculated and presented as Z-scores and T-scores. The OSI Z-score was calculated from the difference between the OSI and the average OSI for healthy individuals of the same age and sex as the participants, and the T-score was calculated from the young adult mean values.

### 2.5 Food frequency questionnaire

The participants completed a food frequency questionnaire during their health checkup (Nakaji et al., 2021). We used the Brief-type self-administered Diet History Questionnaire (BDHQ) to estimate dietary intake during the previous month in healthy adults (Kobayashi et al., 2012; Shiraishi et al., 2017). We adopted energy-adjusted intakes of energy, nutrients, and foods (per day and 1000 kcal) using an ad hoc computer algorithm for further analyses based on the BDHQ validation study (Kobayashi et al., 2012; Kobayashi et al., 2011). The types of dairy foods included low-fat, high-fat (including normal-fat), and total dairy foods, whereas total dairy consumption was the sum of consumption of the low- and high-fat diets. Additionally, dichotomous assignments were applied to habitual and nonhabitual consumers based on the frequency of dairy food consumption. The types of dairy foods, such as milk and yoghurt, were not distinguished using the BDHQ.

### 2.6 Sociodemographic and lifestyle-related questionnaires

Information on sociodemographic and lifestyle-related environmental factors was obtained using a questionnaire that included participant queries (Nakaji et al., 2021). Information on age, sex, and body mass index (BMI) were analyzed for multivariate regressions. The participants who received treatments for osteoporosis were defined as osteoporotic females, and the postmenopausal female participants were demographically defined as females and those older than 54 years old due to missing information on menstrual status.

### 2.7 Statistical analyses

The scarcity of quantitative data on bone turnover markers and the consumption of dairy foods in Japanese adults precluded power calculation in this exploratory study. Thus, the sample size was intrinsically set for all participants in the source cohorts, resulting in approximately 1000 participants, with 10 participants per predictor and 100 possible predictor allowances estimated for this study.

Descriptive statistics were used to describe the participant characteristics. All detected samples were included in the analysis of all variables. Data are presented as means and standard deviations for continuous variables and frequencies and proportions for categorical variables in the baseline characteristics, regardless of the distribution of the data. Differences in characteristics between the groups were investigated using the Mann–Whitney *U* test. Crude correlation analyses were performed to demonstrate convincing correlations among bone biomarkers in the participants. Spearman's correlation coefficient was described in the correlation analysis.

A multivariate linear regression model was developed to identify the associations between dairy consumption and bone turnover markers, including the OSI T-score in the participants, with adjustment for age and sex as confounding factors. For sensitivity analyses, the non-osteoporotic and postmenopausal female participants were included in the secondary analysis.

Statistical analyses were performed using Python (version 3.11.4) and R software (version 4.2.3). Statistical significance was set at *P* < 0.05.

## 3 Results

### 3.1 Participant characteristics, diet, and bone biomarkers

The participants' background characteristics are presented in Table 1 including diets, bone turnover markers and OSI scores. Some participants answered that they did not consume dairy foods and the proportion of no consumption was higher in low-fat dairy than high-fat dairy. For the dichotomous comparison of bone biomarkers and dairy consumption, there was no difference between the participants who took the habitual consumption of dairy foods or not, except for the OSI Z-score in the low-fat dairy and P1NP and ucOC levels and the P1NP/BAP ratio in the high-fat dairy consumptions.

**Table 1:** Background characteristics of all participants and the difference of background characteristics according to dichotomous comparison of dairy consumption (*n* = 1063).

N	All	Low-fat-dairy consumption		High-fat-dairy consumption		Total dairy consumption		*P*-value		
		Yes	No	Yes	No	Yes	No	Low-fat	High-fat	Total-fat
	1063	379	684	757	306	909	154			
***Demographic characteristics***										
Age, Years	56.0 (41.0–66.0)	58.0 (42.0–67.0)	55.5 (41.0–65.0)	56.0 (42.0–66.0)	56.0 (41.0–66.8)	57.0 (42.0–66.0)	53.5 (38.3–64.0)	0.056	0.742	0.037
Male, n (%)	407 (38)	155 (41)	252 (37)	260 (34)	147 (48)	317 (35)	90 (58)	0.193	<0.001	<0.001
BMI, kg/m^2^	22.5 (20.3–24.8)	22.8 (20.5–25.1)	22.3 (20.2–24.6)	22.3 (20.2–24.6)	23.1 (20.6–25.3)	22.5 (20.3–24.7)	23.0 (20.4–24.8)	0.137	0.020	0.500

***Nutrient intake from diet***										
Energy, kcal/d	1769 (1467–2152)	1794 (1492–2160)	1748 (1444–2145)	1775 (1479–2179)	1735 (1419–2084)	1774 (1477–2157)	1724 (1386–2084)	0.153	0.067	0.108
Protein, g/1000 kcal/d	36.4 (32.2–41.4)	37.7 (33.0–42.9)	35.7 (32.0–40.4)	37.0 (33.2–41.4)	35.0 (30.2–41.0)	37.0 (33.2–41.7)	33.0 (28.6–37.7)	<0.001	<0.001	<0.001
Fat, g/1000 kcal/d	27.7 (23.9–31.9)	27.6 (23.2–31.9)	27.8 (24.2–32.0)	28.7 (25.2–32.6)	25.3 (20.6–29.6)	28.3 (24.7–32.2)	24.3 (19.7–28.4)	0.513	<0.001	<0.001
Carbohydrate, g/1000 kcal/d	138.0 (126.3–150.0)	137.8 (127.0–148.9)	138.0 (125.0–150.6)	137.7 (125.9–148.2)	139.5 (126.9–153.4)	137.8 (126.5–148.8)	143.1 (122.7–154.2)	0.958	0.012	0.088

***Food intake from diet***										
Low-fat dairy, g/1000 kcal/d	0 (0–13.8)	36.0 (11.6–75.6)	0 (0–0)	0 (0–6.0)	0 (0–57.7)	0 (0–27.2)	0 (0–0)	<0.001	<0.001	<0.001
High-fat dairy g/1000 kcal/d	22.6 (0–66.5)	6.3 (0–32.8)	37.0 (5.4–76.9)	46.4 (14.9–79.0)	0 (0–0)	33.1 (6.9–73.4)	0 (0–0)	<0.001	<0.001	<0.001
Total dairy, g/1000 kcal/d	47.9 (11.1–84.8)	62.7 (24.7–97.6)	37.0 (5.4–76.9)	61.4 (27.1–90.0)	0 (0–57.7)	61.1 (26.3–89.9)	0 (0–0)	<0.001	<0.001	<0.001

***Bone turnover markers***										
P1NP, μg/L	48.6 (38.0–64.2)	47.1 (37.8–62.4)	49.5 (38.1–64.4)	49.7 (38.1–64.6)	46.0 (37.6–58.7)	48.8 (37.9–64.3)	47.8 (38.5–58.7)	0.410	0.024	0.413
BAP, μg/L	13.2 (10.6–16.7)	13.3 (10.8–16.8)	13.2 (10.5–16.7)	13.2 (10.5–16.8)	13.3 (10.7–16.6)	13.2 (10.5–16.7)	13.5 (10.7–16.7)	0.471	0.906	0.484
NTx, nmol/BCE/L	17.6 (14.4–21.1)	17.6 (14.4–20.9)	17.4 (14.4–21.1)	17.8 (14.5–21.3)	16.9 (14.1–20.5)	17.7 (14.4–21.3)	16.8 (14.3–19.9)	0.951	0.059	0.213
TRACP-5b, mU/dL	367.0 (268.0–493.5)	359.0 (277.5–487.5)	376.0 (261.8–497.3)	376.0 (269.0–497.0)	350.5 (265.0–488.5)	365.0 (269.0–494.0)	373.0 (261.3–489.3)	0.743	0.170	0.765
ucOC, ng/mL	3.3 (2.3–4.4)	3.2 (2.2–4.3)	3.3 (2.3–4.5)	3.4 (2.4–4.5)	2.9 (2.1–4.1)	3.3 (2.3–4.4)	3.0 (2.2–4.3)	0.165	<0.001	0.270
PTH, pg/mL	49.0 (40.0–59.0)	49.0 (39.0–59.0)	49.0 (40.0–60.0)	48.0 (40.0–60.0)	49.0 (41.0–59.0)	48.0 (40.0–59.0)	51.0 (42.0–64.0)	0.709	0.562	0.086
P1NP/BAP	3.7 (3.0–4.4)	3.6 (2.9–4.4)	3.7 (3.0–4.5)	3.7 (3.0–4.5)	3.5 (2.9–4.3)	3.7 (3.0–4.5)	3.5 (2.8–4.3)	0.326	0.020	0.107
NTx/ TRACP-5b	0.047 (0.038–0.0.62)	0.047 (0.039–0.062)	0.047 (0.038–0.060)	0.047 (0.038–0.062)	0.046 (0.039–0.060)	0.047 (0.038–0.062)	0.045 (0.039–0.060)	0.747	0.974	0.422

***Osteo sono assessment index***										
OSI T-score	−0.6 (−1.3–0.3)	−0.6 (−1.2–0.4)	−0.6 (−1.3–0.3)	−0.6 (−1.3–0.4)	−0.6 (−1.2–0.3)	−0.6 (−1.3–0.3)	−0.4 (−1.1–0.4)	0.333	0.478	0.061
OSI Z-score	0.2 (−0.4–0.9)	0.4 (−0.3–1.1)	0.1 (−0.4–0.9)	0.2 (−0.4–0.9)	0.3 (−0.4–0.9)	0.2 (−0.4–0.9)	0.2 (−0.4–0.9)	0.025	0.557	0.777

*P* value was evaluated between consumer and non-consumer using the Mann-Whitney U test.

Data represent median (interquartile range).

BAP, bone-specific alkaline phosphatase; BCE, bone collagen equivalents; OSI, osteo sono assessment index; BMI, body mass index; NTx, N-terminal telopeptide of type I collagen; P1NP, procollagen type I N-terminal peptide; PTH, parathyroid hormone; TRACP-5b, tartrate-resistant acid phosphatase-5b; ucOC, undercarboxylated osteocalcin.

### 3.2 Participant characteristics, diet, and bone biomarkers

The crude correlation analysis between bone turnover markers and OSI is presented in Table 2 and Supplementary Fig. 2 as coefficients and *P*-values. For bone turnover markers, there were significant direct, moderate, and interchangeable correlations among P1NP, BAP, NTx, TRACP-5b, and ucOC, whereas PTH was independent, except for a weak direct correlation with BAP toward bone turnover markers. For the ratio of bone turnover markers, the P1NP/BAP ratio was directly correlated with P1NP, NTx, TRACP-5b, and ucOC and was inversely associated with BAP, PTH, and OSI Z-score in the varying correlation coefficients. The NTx/TRACP-5b ratio was only directly associated with the OSI T-score, whereas other bone turnover markers, including P1NP, BAP, TRACP-5b, and ucOC, were inversely associated with NTx/TRACP-5b. For OSI, both T-score and Z-score were significantly associated inversely with all bone turnover markers, except for PTH.

**Table 2:** Correlation matrix between bone turnover markers and osteo sono assessment index (n = 1063).

Variable	P1NP	BAP	NTx	TRACP-5b	ucOC	PTH	P1NP/BAP	NTx/TRACP-5b	OSI T-score	OSI Z-score
P1NP	–	<0.001	<0.001	<0.001	<0.001	0.228	<0.001	<0.001	<0.001	<0.001
BAP	0.65	–	<0.001	<0.001	<0.001	0.018	<0.001	<0.001	<0.001	<0.001
NTx	0.56	0.51	–	<0.001	<0.001	0.793	<0.001	0.075	<0.001	<0.001
TRACP-5b	0.65	0.69	0.60	–	<0.001	0.667	0.048	<0.001	<0.001	<0.001
ucOC	0.66	0.45	0.49	0.47	–	0.239	<0.001	<0.001	<0.001	<0.001
PTH	−0.04	0.07	−0.01	−0.01	0.04	–	<0.001	0.558	0.623	0.082
P1NP/BAP	0.50	−0.27	0.14	0.06	0.32	−0.12	–	0.250	0.195	0.011
NTx/TRACP-5b	−0.34	−0.42	0.05	−0.73	−0.18	0.02	0.04	–	<0.001	0.753
OSI T-score	−0.23	−0.29	−0.25	−0.38	−0.15	−0.02	0.04	0.28	–	<0.001
OSI Z-score	−0.18	−0.13	−0.18	−0.12	−0.12	0.05	−0.08	0.01	0.79	–

The Spearman correlation coefficients and the P-values represent each cell below and above the diagonal line.

BAP, bone-specific alkaline phosphatase; OSI, osteo sono assessment index; NTx, N-terminal telopeptide of type I collagen; PTH, parathyroid hormone; P1NP, procollagen type I N-terminal peptide; TRACP-5b, tartrate-resistant acid phosphatase-5b; ucOC, undercarboxylated osteocalcin.

### 3.3 Regression analyses in dairy consumption, bone turnover markers, and osteo sono assessment index

The association between dairy consumption and bone turnover markers, including the OSI T-score, was analyzed using multivariate linear regression models with adjustment for age and sex as confounding factors. Data from the multivariate models are summarized in Table 3 and Supplementary Fig. 3. The TRACP-5b and PTH concentrations were inversely associated with low-fat and high-fat dairy consumption in a multivariate model adjusted with age and sex, respectively. Moreover, the ucOC concentration and the P1NP/BAP ratio were directly associated with the high-fat dairy consumption. The OSI T-score and Z-score were directly associated with low-fat dairy consumption. No association was observed between other bone turnover markers and dairy consumption.

**Table 3:** Multivariate linear regression analysis between bone biomarker and dairy consumption (n = 1063).

		β	(95 % CI) SE	Standardized β	*r*^2^	*P*-value
P1NP	Low-fat dairy	−0.026	(−0.07 to −0.019) 0.023	−0.035	0.010	0.254
	High-fat dairy	0.027	(−0.008–0.063) 0.018	0.046	0.011	0.133
	Total dairy	0.008	(−0.023–0.039) 0.016	−0.016	0.009	0.608
BAP	Low-fat dairy	−0.005	(−0.014–0.004) 0.005	−0.031	0.064	0.297
	High-fat dairy	−0.001	(−0.008–0.007) 0.004	−0.005	0.063	0.866
	Total dairy	−0.003	(−0.009–0.004) 0.003	−0.026	0.064	0.382
NTx	Low-fat dairy	−0.007	(−0.021–0.006) 0.007	−0.034	0.013	0.272
	High-fat dairy	0.005	(−0.006–0.016) 0.005	0.028	0.013	0.365
	Total dairy	1.0E-04	(−0.009–0.009) 0.005	0.001	0.012	0.983
TRACP-5b	Low-fat dairy	−0.309	(−0.581 to −0.037) 0.139	−0.063	0.169	0.026
	High-fat dairy	0.086	(−0.133–0.305) 0.111	0.022	0.166	0.440
	Total dairy	−0.085	(−0.276–0.105) 0.097	−0.025	0.166	0.379
ucOC	Low-fat dairy	−0.004	(−0.008–0.001) 0.002	−0.052	0.005	0.091
	High-fat dairy	0.003	(1.6E-04–0.007) 0.002	0.064	0.007	0.040
	Total dairy	0.001	(−0.002–0.004) 0.001	0.019	0.003	0.544
PTH	Low-fat dairy	−0.011	(−0.052–0.030) 0.021	−0.017	0.009	0.590
	High-fat dairy	−0.041	(−0.073 to −0.008) 0.017	−0.076	0.014	0.014
	Total dairy	−0.036	(−0.065 to −0.008) 0.014	−0.078	0.014	0.012
P1NP/BAP	Low-fat dairy	−0.001	(−0.003–0.001) 0.001	−0.020	0.084	0.493
	High-fat dairy	0.002	(0.001–0.004) 0.001	0.077	0.089	0.010
	Total dairy	0.001	(−1.5E-04–0.003) 0.001	0.053	0.086	0.077
NTx/TRACP-5b	Low-fat dairy	1.7E-05	(−1.5E-05–4.8E-05) 1.6E-05	0.029	0.175	0.298
	High-fat dairy	3.9E-06	(−2.1E-05–2.9E-05) 1.3E-05	0.009	0.174	0.758
	Total dairy	1.1E-05	(−1.1E-05–3.3E-05) 1.1E-05	0.028	0.175	0.320
OSI T-score	Low-fat dairy	0.002	(1.2E-04–0.004) 0.001	0.057	0.226	0.037
	High-fat dairy	3.6E-05	(−0.002–0.002) 0.001	0.001	0.223	0.963
	Total dairy	0.001	(−3.2E-04–0.002) 0.001	0.041	0.224	0.136
OSI Z-score	Low-fat dairy	0.002	(3.8E-04–0.005) 0.001	0.071	0.011	0.021
	High-fat dairy	4.0E-05	(−0.002–0.002) 0.001	−0.001	0.006	0.962
	Total dairy	0.001	(−2.9E-04–0.003) 0.001	0.049	0.008	0.116

Models were adjusted for age and sex. BAP, bone-specific alkaline phosphatase; OSI, osteo sono assessment index; NTx, N-terminal telopeptide of type I collagen; PTH, parathyroid hormone; P1NP, procollagen type I N-terminal peptide; TRACP-5b, tartrate-resistant acid phosphatase-5b; ucOC, undercarboxylated osteocalcin.

For sensitivity analysis, further associations were explored in postmenopausal female participants, and comparisons were performed among participants with respect to osteoporosis treatment status. In the analyses of postmenopausal female participants, there were statistically significant results in all participants, including TRACP-5b, OSI T-score, and Z-score toward low-fat dairy consumption and ucOC and P1NP/BAP toward high-fat dairy consumption in Table 4. In addition, the inverse association between ucOC and low-fat dairy consumption and the direct association between P1NP/BAP and total dairy consumption were significant in postmenopausal female participants, whereas other associations found in the entire study population were not statistically significant in postmenopausal female participants. Meanwhile, there were significant differences in P1NP, P1NP/BAP ratio, NTx/TRACP-5b ratio, and OSI T-score for the treatment status of osteoporosis in Table 5.

**Table 4:** Multivariate linear regression analysis between bone biomarker and dairy consumption in postmenopausal female (n = 378).

		β	(95 % CI) SE	Standardized β	*r*^2^	*P*-value
P1NP	Low-fat dairy	−0.041	(−0.097–0.015) 0.028	−0.072	0.068	0.150
	High-fat dairy	0.045	(−0.006–0.096) 0.026	0.086	0.070	0.084
	Total dairy	0.008	(−0.038–0.055) 0.023	0.018	0.063	0.719
BAP	Low-fat dairy	−0.009	(−0.023–0.005) 0.007	−0.064	0.024	0.211
	High-fat dairy	−0.002	(−0.015–0.011) 0.007	−0.017	0.020	0.746
	Total dairy	−0.008	(−0.020–0.004) 0.006	−0.068	0.024	0.186
NTx	Low-fat dairy	−0.012	(−0.028–0.003) 0.008	−0.080	0.015	0.119
	High-fat dairy	0.009	(−0.005–0.023) 0.007	0.065	0.013	0.206
	Total dairy	−0.001	(−0.014–0.012) 0.006	−0.008	0.008	0.879
TRACP-5b	Low-fat dairy	−0.468	(−0.884- -0.053) 0.211	−0.114	0.015	0.027
	High-fat dairy	0.230	(−0.154–0.614) 0.195	0.061	0.006	0.240
	Total dairy	−0.134	(−0.478–0.211) 0.175	−0.039	0.004	0.446
ucOC	Low-fat dairy	−0.006	(−0.012- -0.001) 0.003	−0.114	0.019	0.027
	High-fat dairy	0.006	(0.001–0.011) 0.003	0.125	0.022	0.015
	Total dairy	0.001	(−0.004–0.005) 0.002	0.018	0.007	0.724
PTH	Low-fat dairy	0.008	(−0.036–0.052) 0.022	0.018	0.012	0.725
	High-fat dairy	−0.025	(−0.065–0.015) 0.021	−0.062	0.015	0.225
	Total dairy	−0.015	(−0.051–0.022) 0.018	−0.041	0.013	0.427
P1NP/BAP	Low-fat dairy	−0.001	(−0.003–0.002) 0.001	−0.020	0.037	0.699
	High-fat dairy	0.004	(0.001–0.006) 0.001	0.150	0.059	0.003
	Total dairy	0.003	(4.1E-04-0.005) 0.001	0.118	0.050	0.019
NTx/TRACP-5b	Low-fat dairy	2.1E-05	(−1.0E-05- 5.3E-05) 1.6E-05	0.068	0.005	0.185
	High-fat dairy	−8.7E-06	(−3.8E-05- 2.1E-05) 1.5E-05	−0.030	0.001	0.558
	Total dairy	7.6E-05	(−1.9E-05- 3.3E-05) 1.3E-05	0.029	0.001	0.571
OSI T-score	Low-fat dairy	0.002	(1.9E-04–0.004) 0.001	0.107	0.086	0.032
	High-fat dairy	−2.1E-04	(−0.002–0.002) 0.001	−0.011	0.074	0.824
	Total dairy	0.001	(3.3E-04–0.003) 0.001	0.078	0.080	0.117
OSI Z-score	Low-fat dairy	0.003	(2.5E-04-0.005) 0.001	0.112	0.016	0.030
	High-fat dairy	−2.3E-04	(−0.002–0.002) 0.001	−0.011	0.003	0.833
	Total dairy	0.002	(−3.4E-04-0.003) 0.001	0.082	0.010	0.109

Models were adjusted for age. BAP, bone-specific alkaline phosphatase; OSI, osteo sono assessment index; NTx, N-terminal telopeptide of type I collagen; PTH, parathyroid hormone; P1NP, procollagen type I N-terminal peptide; TRACP-5b, tartrate-resistant acid phosphatase-5b; ucOC, undercarboxylated osteocalcin.

**Table 5:** The difference of background characteristics according to dichotomous comparison of treatment status for osteoporosis.

N	Non-treatment 1034	Osteoporosis 29	*P*-value
***Demographic characteristics***			
Age, years	56.0(41.0–65.0)	75.0(67.0–76.0)	<0.001
Male, n (%)	406(39)	1(3)	<0.001
BMI, kg/m^2^	22.5(20.3–24.8)	21.2(19.1–23.3)	0.025

***Nutrient intake from diet***			
Energy, kcal/d	1770(1467–2154)	1682(1447–1930)	0.229
Protein, g/1000 kcal/d	36.3(32.2–41.1)	42.0(37.0–45.5)	0.001
Fat, g/1000 kcal/d	27.7(23.9–31.9)	28.9(25.7–32.1)	0.781
Carbohydrate, g/1000 kcal/d	137.9(125.9–150.1)	138.2(131.2–149.6)	0.402

***Food intake from diet***			
Low-fat dairy, g/1000 kcal/d	0(0–13.9)	0(0–7.1)	0.782
High-fat dairy, g/1000 kcal/d	22.6(0–66.6)	20.2(0–60.0)	0.904
Total dairy, g/1000 kcal/d	48.3(11.1–84.7)	37.9(8.0–86.0)	0.857

***Bone turnover marker***			
P1NP, μg/L	49.0(38.1–64.3)	37.8(26.5–43.7)	<0.001
BAP, μg/L	13.3(10.6–16.7)	11.6(10.2–15.4)	0.133
NTx, nmol/BCE/L	17.6(14.4–21.1)	14.9(13.0–19.7)	0.082
TRACP-5b, mU/dL	365.0(265.3–495.5)	409.0(339.0–475.0)	0.324
ucOC, ng/mL	3.3(2.3–4.4)	3.0(2.0–4.4)	0.328
PTH, pg/mL	49.0(40.0–59.0)	56.0(47.0–65.0)	0.073
P1NP/BAP ratio	3.7(3.0–4.4)	2.9(2.3–4.4)	0.010
NTx/TRACP-5b ratio	0.047(0.039–0.062)	0.039(0.033–0.049)	0.002

***Osteo sono assessment index***			
OSI T-score	−0.6(−1.3–0.4)	−1.6(−2.0–1.2)	<0.001
OSI Z-score	0.2(−0.4–1.0)	0.1(−0.5–0.6)	0.340

*P* value was evaluated between non-osteoporosis populations and osteoporosis populations using the Mann-Whitney U test.

Data represent median (interquartile range).

BAP, bone-specific alkaline phosphatase; BCE, bone collagen equivalents; OSI, osteo sono assessment index; BMI, body mass index; NTx, N-terminal telopeptide of type I collagen; P1NP, procollagen type I N-terminal peptide; PTH, parathyroid hormone; TRACP-5b, tartrate-resistant acid phosphatase-5b; ucOC, undercarboxylated osteocalcin.

## 4 Discussion

This study described the association between dairy consumption and bone turnover markers and OSI in Japanese adults. High dairy food consumption was partly associated with reduced bone turnover markers, classified as bone formation and resorption markers. As the IHPP had a relatively high proportion of elderly female participants (*n* = 378, 35.5 %), the baseline characteristics appeared to reflect a rapid increase in bone turnover after menopause (Garnero et al., 1996). The bone formation index, calculated as the P1NP/BAP ratio, increased with increased consumption of high-fat dairy products, whereas the bone resorption index, calculated as the NTx/TRACP-5b ratio, was not affected by dairy consumption. These results imply that dairy consumption is preferable for stimulating bone formation, which is often observed in postmenopausal females. In addition, participants' backgrounds appeared to be different from the consumers of low-fat and high-fat dairy foods (Table 1). The dietary fatty acid profile is associated with bone density among saturated fatty acids, monounsaturated fatty acids, and polyunsaturated fatty acids (Corwin et al., 2006), while full-fat or low-fat dairy is not associated with all-cause mortality or cardiovascular outcomes in healthy people (Giosuè et al., 2022). Lipid-soluble micronutrients like carotenoids also modulate bone metabolism. Combined, dietary patterns associated with dairy fat may be involved in bone turnover markers and bone mineral density.

Meanwhile, the increased consumption of dairy foods was directly associated with the level of ucOC, a nutritional marker of vitamin K status. Vitamin K involves in the carboxylation of osteocalcin and thus ucOC status is vitamin K-dependent (Gundberg et al., 2012). In the Japanese diet, *natto*, a fermented soybean, contains considerable vitamin K. As natto contains >100 times more vitamin K2 than various cheeses, it is a major source of dietary vitamin K in Japan (Katsuyama et al., 2002). Indeed, the mean daily intakes of natto and vitamin K were high but not significant in consumer vs non-consumer for any types of dairy foods (natto, 12.6 ± 11.3 vs 10.4 ± 8.4 g/1000 kcal; vitamin K, 172 ± 91 vs 162 ± 73 μg/1000 kcal for consumer vs non-consumer). High-fat dairy remained borderline significant in the direct association with ucOC in another multivariate model with an adjustment for vitamin K, age, and sex (Standardized β = 0.06, *P* = 0.052 for high-fat dairy). Dietary natto intake is associated with reduced bone loss in postmenopausal females in Japan (Fu et al., 2017), while US dairy products are a natural source of multiple forms of vitamin K (Ikeda et al., 2006). A cohort study found a direct association between natto intake and the rates of changes in BMD at the femoral neck and the distal third of the radius in postmenopausal females in Japan (Fu et al., 2017). Considering the nature of dietary exposure to vitamin K and its nutritional fortification, dairy foods are a promising source of vitamin K in Japan.

In a Japanese population-based cross-sectional study, PTH was directly associated with age in both sexes, whereas BAP and TRACP-5b were directly associated with age only in female participants (Kikuchi et al., 2021). Levels of 1,25-dihydroxyvitamin D (1,25(OH)2D) are tightly regulated by parathyroid hormone (PTH), phosphate and calcium. (Haarburger et al., 2009). We demonstrated that low-fat dairy consumption was inversely associated with TRACP-5b and directly associated with OSI T-scores. High-fat and total dairy consumption were inversely associated with PTH in multivariate linear regression models adjusted for age and sex. A French intervention trial found that dairy foods fortified with vitamin D and calcium intake had significantly lower concentrations of TRACP-5b and PTH, with increased serum 25-hydroxyvitamin D levels, compared with unfortified dairy foods (Bonjour et al., 2013; Bonjour et al., 2015). Dairy foods are a major source of dietary calcium, and PTH stimulates calcium release from large calcium stores, such as bones, into the bloodstream (Kužma et al., 2021). Our results suggest that dairy intake reflects reduced parathyroid levels via calcium homeostasis during bone metabolism in a real-world setting. Meanwhile, the intake of low-fat dairy foods at 4–5 servings/day or calcium and vitamin D during a 6-month intervention resulted in better weight loss and bone mineral density (BMD) compared with hypocaloric diets in overweight and obese Caucasian early postmenopausal female participants (Ilich et al., 2019). In a cross-sectional study, habitual milk intake is associated with low bone turnover and high areal BMD in community-dwelling elderly Japanese males (Sato et al., 2015). Accordingly, low-fat dairy consumption may potentially increase BMD by improving body composition, although the cause–effect relationship should be warranted and the influence of other confounders, such as ethnicity and exercise, should also be considered (Schini et al., 2023).

Considering bone turnover markers, P1NP and C-terminal cross-linked telopeptide of type I collagen (CTx) are the serum biomarkers recommended by the International Osteoporosis Foundation for predicting fracture risk and monitoring osteoporosis treatment (Vasikaran et al., 2011). TRACP-5b reflects the abundance of osteoclasts, as this bone-specific acid phosphatase enzyme is secreted from osteoclasts, whereas NTx and CTx are degradation products of collagen released only by the degradation of the bone matrix (Henriksen et al., 2007; Seibel, 2005). TRACP-5b has a similar diagnostic accuracy to PINP and CTx, resulting in the proposal of TRACP-5b for monitoring patients on oral bisphosphonates, such as zoledronate (Gossiel et al., 2022). The highest correlation coefficients were observed in TRACP-5b toward P1NP, BAP, and NTx in the correlation matrix (Table 2). As the intake of food, glucose, fat, and protein reduces CTx independently from age and sex (Bjarnason et al., 2002), TRACP-5b may be more suitable to evaluate the association between dairy consumption and bone resorption markers than the biomarkers of collagen degradation products, such as CTx and NTx.

The association observed in this study could be partly explained by the bioactive proteins and peptides identified and investigated for their effects on bone health in bovine milk and dairy foods. Milk basic protein is a fractionated protein mixture obtained via cation exchange and contains a range of basic bioactive proteins, such as lactoferrin, lactoperoxidase, cystatin, kininogen fragment, high mobility group-like protein, angiogenin, and transforming growth factor-beta (Kawakami, 2005; Morita et al., 2008; Morita et al., 2012; Ono-Ohmachi et al., 2019). These bioactive proteins modulate bone metabolism, including stimulating bone formation through osteoblasts and reducing bone resorption through osteoclasts in in vitro and in vivo studies and human clinical studies (Yamamura et al., 2002; Aoe et al., 2005; Uenishi et al., 2007). Among the milk-derived peptides, casein phosphopeptides facilitate calcium absorption (Tenenbaum et al., 2022). Accordingly, habitual consumption of dairy foods may contribute to the nutritional supplementation of these bioactive milk proteins for bone health, even in limited dairy consumption, like in Japan, although the effective dosage and bioactivity remain to be explained in detail.

This study has some potential limitations. First, the cross-sectional design and potential bias of the study participants affected the results. Certainly, some of the associations were found in older (age ≥55 years) female participants in the stratified sensitivity analysis, although the associations were adjusted for age and sex in the multivariate models. The causality of these associations was not warranted owing to the cross-sectional nature of this study. Second, the OSI obtained from the QUS imaging modality did not accurately estimate the true BMD outcomes from X-ray-based techniques, such as dual X-ray absorptiometry scans. Third, a food frequency questionnaire is easily applied to medical checkups for estimating dietary profiles but has concurrent limitations on the precision and accuracy of their estimates compared with the golden standard method, 24-h dietary recall. Further improvement of these limitations warrants the observations found in this study. As 154 participants (14 % of the study participants) answered themselves as non-consumer of any type of dairy foods (Table 2) and BDHQ cannot distinguish normal nor high-fat dairy foods, an alternative approach would be needed to describe the detailed information on dairy consumption in Japanese.

In conclusion, dairy consumption is partly associated with bone turnover biomarkers and OSI in Japanese adults, mainly postmenopausal females. Our results suggest that dairy consumption in Japan partly reflects bone turnover markers, including the types of dairy foods, and that sociodemographic status has considerable potential for maintaining bone health. Further follow-up investigations and intervention studies are required to determine the clinical implications of these associations in bone health.

## Sources of support

The Japanese Science and Technology Agency and Megmilk Snow Brand Co., Ltd. supported this study.

## Funding

This work was supported by the Japan Science and Technology Agency (grant numbers JPMJCE1302, JPMJCA2201, and JPMJPF2210) and Megmilk Snow Brand Co., Ltd. (approval number 2022–30444).

## CRediT authorship contribution statement

**Ayatake Nakano:** Conceptualization, Formal analysis, Investigation, Data Curation, Writing - original draft, Visualization. **Hiroshi M. Ueno:** Conceptualization, Methodology, Formal analysis, Investigation, Resources, Data Curation, Writing - original draft, Supervision, Project administration, Funding acquisition. **Daisuke Kawata:** Conceptualization, Investigation, Writing - review & editing. **Yota Tatara:** Resources, Investigation, Writing - review & editing. **Yoshinori Tamada:** Resources, Investigation, Writing - review & editing, Data curation, Project administration, Funding acquisition. **Tatsuya Mikami:** Resources, Investigation, Writing - review & editing, Supervision, Project administration, Funding acquisition. **Koichi Murashita:** Resources, Investigation, Writing - review & editing, Supervision, Project administration, Funding acquisition. **Shigeyuki Nakaji:** Resources, Investigation, Writing - review & editing, Supervision, Project administration, Funding acquisition. **Ken Itoh:** Resources, Investigation, Writing - review & editing, Supervision, Project administration, Funding acquisition.

## Declaration of competing interest

This study was supported by the 10.13039/501100002241Japan Science and Technology Agency (grant numbers JPMJCE1302, JPMJCA2201, and JPMJPF2210) and Megmilk Snow Brand Co., Ltd. (approval number 2022-30444). A.N., D.K., and H.M.U. are employees of the Megmilk Snow Brand. The Megmilk Snow Brand funded the Department of Precision Nutrition for Dairy Foods at Hirosaki University and provided human resources and infrastructure for this study. The Megmilk Snow Brand manufactures and sells dairy foods in Japan and some Asian and Oceanian countries. The other authors declare no conflicts of interest.

## Data Availability

Data, including analytical codes, cannot be shared publicly because of ethical concerns. Data are available from the Hirosaki University COI Institutional Data Access/Ethics Committee (contact via e-mail: coi@hirosaki-u.ac.jp) for researchers who meet the criteria for data access. Researchers need to be approved by the research ethics review board of the organization of their affiliations.

## Appendix A Supplementary data

Supplementary figures Image 1
