# Supplementary material for: Dairy consumption, bone turnover biomarkers, and osteo sono assessment index in Japanese adults: A cross-sectional analysis of data from the Iwaki Health Promotion Project
Source: Bone Rep. 2024 Apr 29;21:101770. doi: 10.1016/j.bonr.2024.101770 (PMC11087920; doi:10.1016/j.bonr.2024.101770)
Supplement: Supplementary file 1 — Supplementary figures [file mmc1.docx]

**Supplemental data for publication**

**SUPPLEMENTARY FIGURE 1**

Flowchart showing participant disposition and grouping in the study.

Participants enrollment

n = 1113

53 participants did not have records due to missing data.

・ BDHQ, n=47

・ OSI T-score, n=5

・ P1NP, n=1

2 participants were excluded

・ Due to the records of outrange for the calibration curve in ucOC.

Participants analyzed

n = 1063

・ Participants who receive the treatment of osteoporosis, n=29

・ Pre-defined postmenopausal females of age≧55, n=378

Abbreviations: BDHQ, Brief-type self-administered Diet History Questionnaire; OSI, osteo sono assessment index; ucOC, undercarboxylated osteocalcin; total P1NP, total procollagen type I N-terminal peptide

**SUPPLEMENTARY FIGURE 2**

Correlation matrix between bone turnover markers and osteo sono assessment index

This heat map shows the results of the Spearman correlation coefficient between the x- and y-axis variables. ^*^, ^**^, and ^***^ represent significant correlations at *P*<0.05, <0.01, and <0.001, respectively. BAP, bone-specific alkaline phosphatase; OSI, osteo sono assessment index; NTx, N-terminal telopeptide of type I collagen; PTH, parathyroid hormone; P1NP, procollagen type I N-terminal peptide; TRACP-5b, tartrate-resistant acid phosphatase-5b; ucOC, undercarboxylated osteocalcin.

**
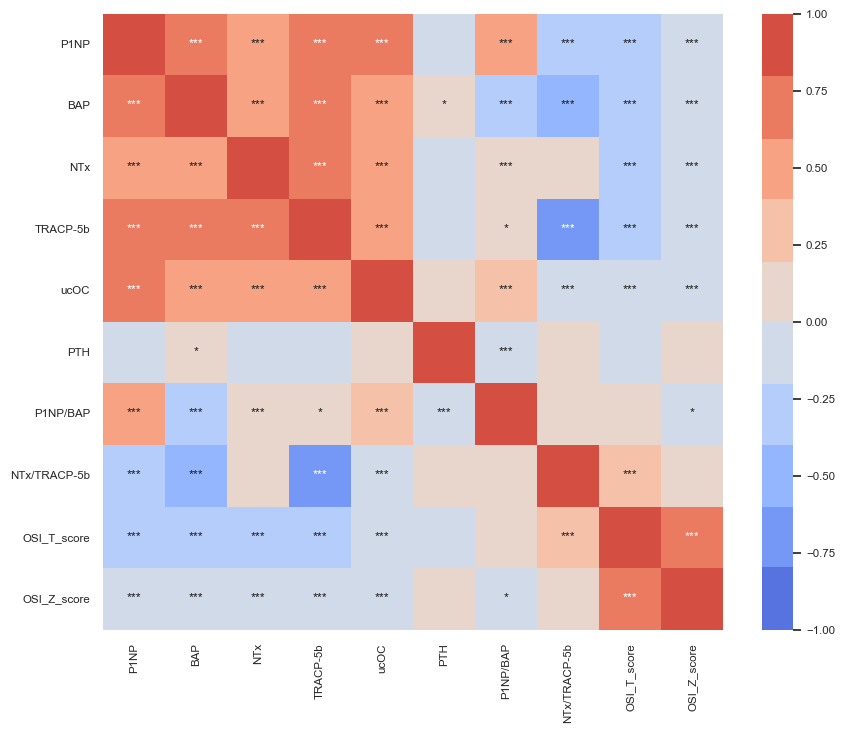
**

**SUPPLEMENTARY FIGURE 3**

Regression coefficient between dairy consumption and bone biomarkers in multivariate linear regression analysis. Each column on the x-axis represents a variable of low for low-fat dairy consumption, high for high-fat dairy consumption, and total for total-fat dairy consumption. This heat map represents the results of the multivariate linear regression coefficient between the x- and y-axis variables, adjusted for age and sex. ^*^ and ^**^ represent significant correlations at *P*<0.05 and <0.01, respectively. BAP, bone-specific alkaline phosphatase; OSI, osteo sono assessment index; NTx, N-terminal telopeptide of type I collagen; PTH, parathyroid hormone; P1NP, procollagen type I N-terminal peptide; TRACP-5b, tartrate-resistant acid phosphatase-5b; ucOC, undercarboxylated osteocalcin.

**
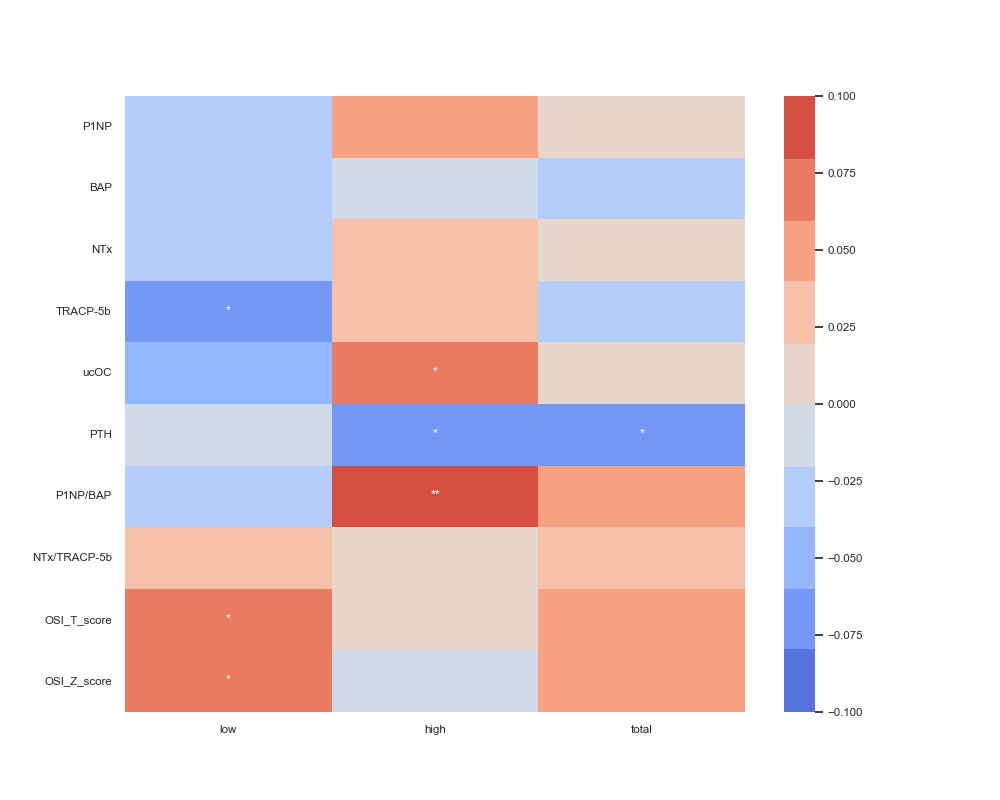
**
